# Effects of Ketamine, S-Ketamine and MK 801 on Integrin Beta-3-mediated Cell Migration in Pancreatic Carcinoma

**DOI:** 10.26502/jcsct.5079183

**Published:** 2022-12-22

**Authors:** Manuela Malsy, Veronika Hofer, Stephan Schmidbauer, Bernhard Graf, Anika Bundscherer

**Affiliations:** 1 Department of Anesthesiology, University Medical Center Regensburg, Germany

**Keywords:** Integrin Beta-3, Ketamine, Migration, MK 801, Pancreatic Cancer, S-Ketamine

## Abstract

**Introduction:**

Pancreatic ductal adenocarcinoma is one of the most aggressive malignancies in humans. The main reason for its unfavourable prognosis is the combination of rapid tumour growth, early-onset metastasis and currently still inadequate diagnostic and therapeutic options. Thus, only very few patients are eligible for radical resection of the primary tumour as the only curative treatment option available so far. In the perioperative period, tumour progression and metastasis are facilitated by the activation of key signalling pathways and the altered regulation of transcription factors. Various tumour entities have shown increased expression of the integrin-3 receptor subunit, which correlates with more rapid tumour progression and metastasis through advanced migration, invasion and proliferation. The influence of perioperative medication and postoperative pain management remains unclear. To investigate the effects of ketamine, s-ketamine and MK 801 on integrin beta-3-mediated cell migration in pancreatic cancer cells *in vitro*.

**Methods:**

The effects of ketamine, s-ketamine and MK 801 on integrin beta-3 expression were investigated with immunoblot. Cell migratory potentials were analysed using a Cell Migration Assay Kit with a Boyden chamber, in which cells migrate through a semipermeable membrane under different stimuli.

**Results:**

Stimulation with ketamine and MK 801 significantly promoted migration in pancreatic cancer cells, increasing the expression of integrin beta-3.

**Conclusion:**

Novel therapeutic approaches target the effective modulation of specific signalling and transcription pathways. The prerequisite for such ‘target therapies’ is comprehensive knowledge about the respective carcinogenesis. Further studies are required to identify the underlying disease mechanisms of pancreatic carcinoma.

## 1. Introduction

Pancreatic ductal adenocarcinoma is one of the most aggressive types of malignant tumours in humans. With increasing incidence rates in western industrialised countries and a 5-year survival rate of less than 10% after diagnosis [1], pancreatic carcinoma is the fourth most common tumour-related cause of death in Germany and other western countries [2]. Although some progress in the molecular and biological understanding of pancreatic carcinoma has been made in recent years [3], patients’ survival rates have not significantly improved. Long-term survival is still almost impossible [4]. The main reason for the unfavourable prognosis of pancreatic carcinoma is the combination of rapid tumour growth, early-onset metastasis and currently still inadequate diagnostic and therapeutic options [5]. Thus, only very few patients are eligible for radical resection of the primary tumour as the only curative treatment option available so far [6]. Recent studies have shown that the perioperative period is a particularly vulnerable phase, in which tumour progression and metastasis are facilitated by the constitutive activation of key signalling pathways and the altered regulation of transcription factors [7]. Various tumour entities have shown increased expression of the integrin-3 receptor subunit on tumour cells and vascular endothelial cells [8], which correlates with more rapid tumour progression and metastasis through increased migration, invasion and proliferation [9]. The extent of the influence of medication administered in the perioperative setting or as postoperative pain therapy remains unclear. Aim of this study is to investigate the effects of ketamine, s-ketamine and MK 801 on integrin beta-3-mediated migration of pancreatic cancer cells *in vitro*.

## 2. Material and Methods

### 2.1 Cell Lines

The human pancreatic cancer cell lines PaTu 8988t and PANC-1 were obtained from Professor Ellenrieder (Philipps University of Marburg, Germany). PaTu 8988t and PANC-1 cells were maintained in Dulbecco’s modified Eagle’s medium (Sigma-Aldrich) supplemented with 10% foetal calf serum (Sigma-Aldrich) and 5% Myco Zap (Lonza Verviers SPRL). Cells were cultured in humidified CO_2_ atmosphere (5%) at 37°C and maintained in monolayer culture. Experiments were done with cells at ~70–80% confluence.

### 2.2 Reagents

Commercially available ketamine, s-ketamine and MK 801 were purchased from Sigma-Aldrich. Final concentrations were obtained by diluting drugs in standard growth media. All solutions were prepared freshly prior to use.

### 2.3 Cell Lysate

Cells were washed twice with cold DPBS and re-suspended in RIPAE buffer (5 mL Triton X100, 190 mg EDTA, 0.5 g SDS, 2.5 g deoxycholic acid, 500 mL DPBS, proteinase inhibitors) for 15 min and centrifuged at 13.000 rpm for 30 min. Supernatants were transferred to new cups and incubated on ice.

### 2.4 Subcellular Fractionation and Immunoblotting

Cells were washed twice with cold DPBS and collected through centrifugation at 4000 rpm at 4°C for 10 min. Lysates were then re-suspended in RIPAE buffer (5 mL Triton X100, 190 mg EDTA, 0.5 g SDS, 2.5 g deoxycholic acid, 500 mL DPBS, proteinase inhibitors) for 15 min and centrifuged at 13.000 rpm for 30 min. Supernatants were transferred to new cups and incubated on ice. For Western blotting, 30 μg protein extracts were analysed with SDS-PAGE and blotted onto nitrocellulose. Upon protein extraction and gel transfer, membranes were washed in TBS washing buffer and incubated with peroxidase-conjugated secondary antibodies. Immunoreactive proteins were visualised by means of an enhanced chemi-luminescence detection system (Western Blotting Detection Reagent, GE Healthcare). For immunoblotting, membranes were probed with antibodies against integrin beta-3 (cell signalling) and ß-actin (Sigma-Aldrich).

### 2.5 Cell Migration Assay

Cell migratory potentials were evaluated using a Cell Migration Assay Kit (abcam). The test includes a Boyden chamber, in which the cells migrate through a semipermeable membrane under different stimuli. In brief, cells were treated with 0 μM, 10 μM, 100 μM or 1000 μM ketamine, with 0 μM, 10 μM, 100 μM or 1000 μM s-ketamine or with 0 μM, 10 μM, 100 μM or 1000 μM MK 801 in a serum-free medium for 2 h. Afterwards, 200.000 cells of the human pancreatic cancer cell lines PaTu 8988t or PANC-1 were placed into the upper Boyden chamber, and a stimulant was pitted into the lower chamber. The chambers were incubated at 37°C for 24 h. The migrating cells passed through the semipermeable membrane and migrated into the bottom chamber or adhered to the bottom of the upper chamber. After dismantling, cell migration was directly analysed by means of reading fluorescence (Ex/Em = 530/590 nm) in a plate reader. All tests were done with three wells per treatment group and performed as two independent experiments.

### 2.6 Statistical Analysis

Data are presented as mean ± SD. The non-parametric Mann Whitney U-test was used for statistical evaluation of the data. P-values of <0.05 were considered significant. IBM SPSS Statistics (Vs. 26; IBM New York, US) and Excel Vs. 2019 (Microsoft, Redmond, USA) packages were employed for statistical analysis.

## 3. Results

### 3.1 Endogenous Proof of Integrin Beta-3 Expression in the Pancreatic Cancer Cell Lines PaTu 8988t and PANC-1 after Stimulation with Ketamine, S-Ketamine and MK 801

**Figure 1:**
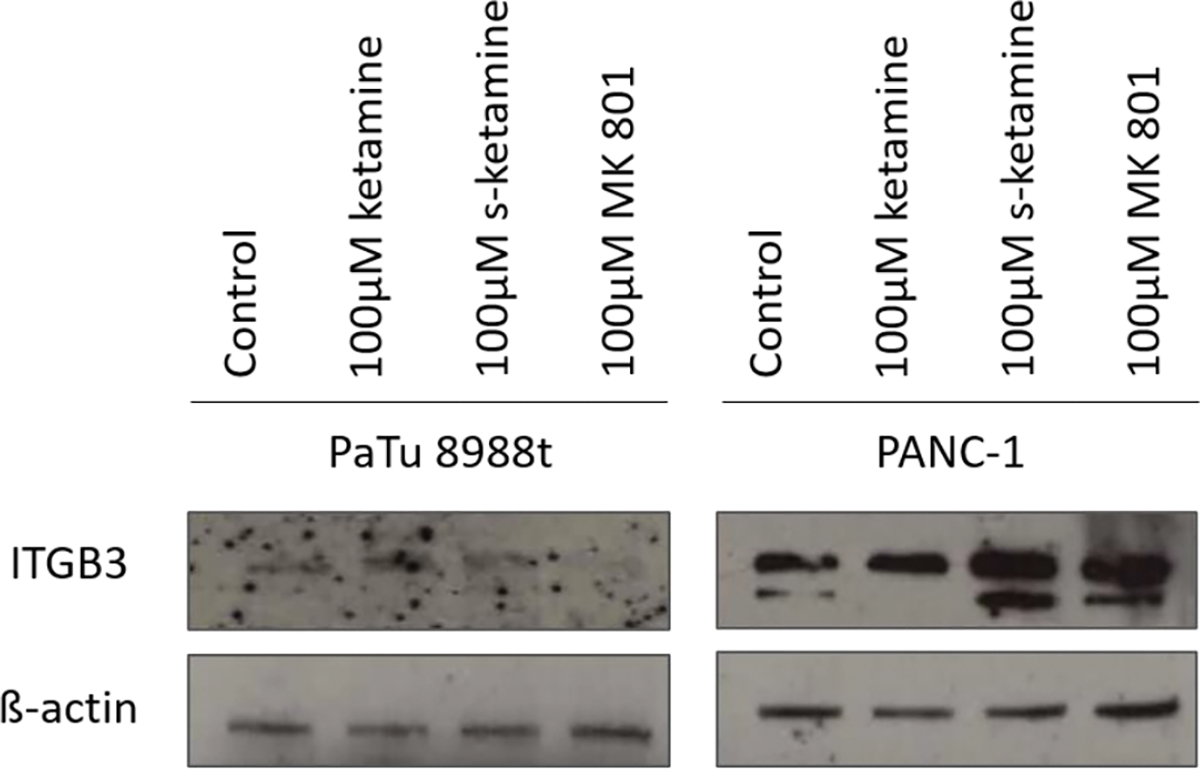
Immunoblotting and proof of the endogenic expression of integrin beta-3 in the pancreatic cancer cell line PaTu 8988t und PANC-1 after stimulation with 100 μM ketamine, 100 μM s-ketamine or 100 μM MK 801.

The first aim was to obtain evidence for the expression of integrin beta-3 in pancreatic cancer cells (Figure 1). Expression of integrin beta-3 was very weak in the pancreatic cancer cell line PaTu 8988t but much stronger in PANC-1 (Column 1). Stimulation with 100 μM ketamine, 100 μM s-ketamine or 100 μM MK 801 increased expression of integrin beta-3 in both pancreatic cancer cell lines compared to unstimulated control (Column 1). The endogenous expression of ß-actin serving as loading control can be seen in the lower blot (Column 2).

### 3.2 Analysis of Migration in Pancreatic Cancer Cells

**Figure 2 (a,b):**
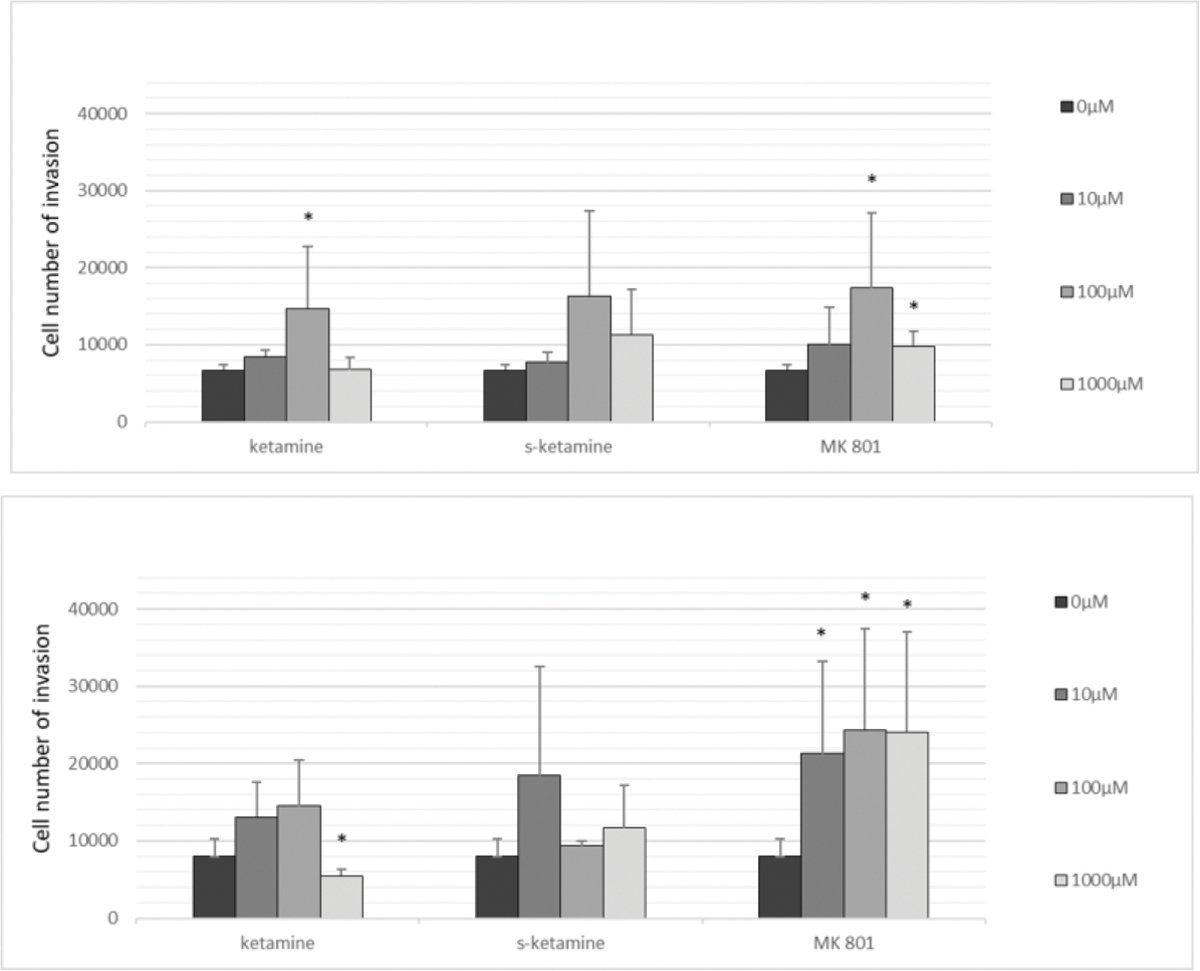
The effects of ketamine, s-ketamine and MK 801 on cell migration in the pancreatic cell lines PaTu 8988t (a) and PANC-1 (b) *in vitro*. Cell migration was quantified by means of a Boyden chamber, in which cells migrate through a semipermeable membrane under different stimuli. (*) indicates statistical significance at p <0.05 compared to untreated control.

A Cell Migration Assay Kit was used to determine whether stimulation with ketamine, s-ketamine or MK 801 induced cell migration. The test includes a Boyden chamber, in which cells migrate through a semipermeable membrane under different stimuli. For this purpose, the pancreatic cancer cell lines PaTu 8988t and PANC-1 were treated with 0 μM, 10 μM, 100 μM or 1000 μM ketamine, with 0 μM, 10 μM, 100 μM or 1000 μM s-ketamine or with 0 μM, 10 μM, 100 μM or 1000 μM MK 801 in a serum-free medium for 2 h. After 24 h incubation, cell migration was directly analysed by means of reading fluorescence in a plate reader. The pancreatic cancer cell lines PaTu 8988t (a) and PANC-1 (b) showed increased cell migration after stimulation with 100 μM and 1000 μM MK 801 compared to untreated control; the cell line PANC-1 also showed an additive increase in cell migration after stimulation with 10 μM MK 801 (Figure 2). In the cell line PaTu 8988t, stimulation with 10 μM and 100 μM ketamine also led to a statistically significant increase in cell migration (Figure 2a). In the pancreatic cancer cell line PANC-1, stimulation with 1000 μM ketamine significantly inhibited cell migration (Figure 2b).

## 4. Discussion

**Figure 3 (a + b):**
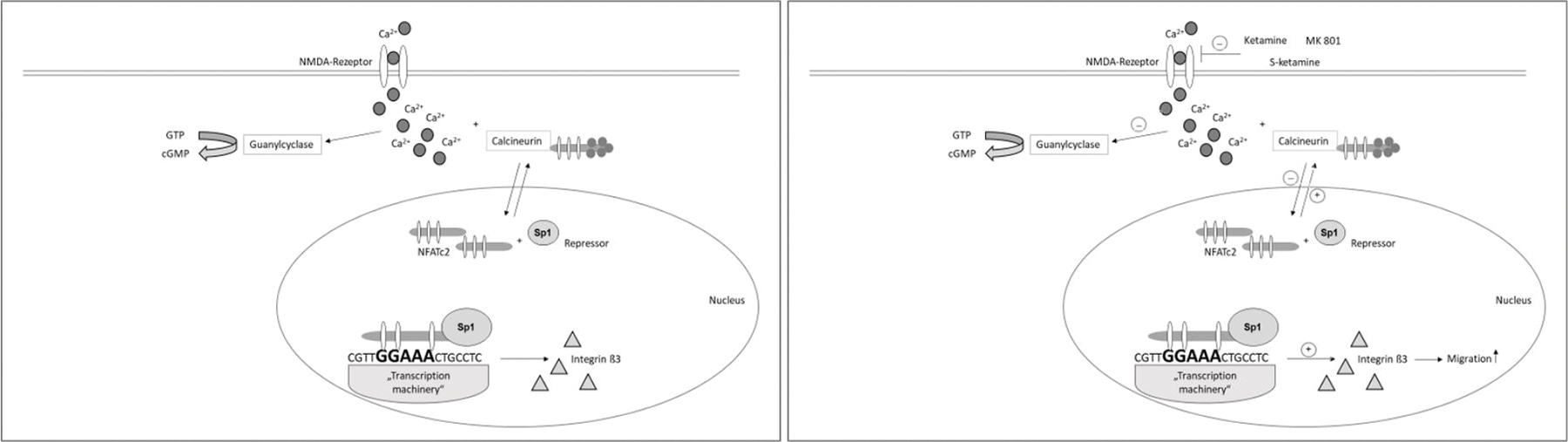
Potential molecular biological mechanism of the effect of ketamine, s-ketamine and MK 801 on integrin beta-3-mediated migration in pancreatic carcinoma.

In carcinogenesis, the metastasis of tumour cells represents the endpoint of a multi-step process [10]. Metastases develop when cancer cells detach from the primary tumour, migrate with blood or lymph and relocate and multiply in other tissues. Molecular biological analyses have shown loss or inactivation of cell-cell or cell-matrix adhesion molecules [11]. Integrins bind to various proteins of the extracellular matrix and mediate bidirectional signal transduction. Integrins are transmembrane heterodimeric glycoproteins, which consist of 18 different alpha subunits and 8 different beta subunits, forming a total of 24 integrins [12]. Integrins typically occur on the surface of cell membranes only for a limited period of time [13]. The presence or absence of integrins has a huge influence on the capacity of malignant tumours to invade and destroy local tissue, thus also on the metastasis of tumour cells [14]. A key factor in the carcinogenesis of pancreatic carcinoma is the family of NFAT (nuclear factor of activated T-cells) transcription factors [15]. Preliminary work has shown that NFATc2 interacts with other transcriptional partners, such as the oncogenic protein Sp1 in pancreatic cancer, and that carcinogenic effects are caused by the interaction of NFATc2 and Sp1 [16]. The application of ketamine or s-ketamine seems to inhibit this activation cascade [17]. As N-methyl-D-aspartate (NMDA) receptor antagonists, ketamine, s-ketamine and MK 801 bind at the phencyclidine binding site inside the NMDA channel, thereby inhibiting the action of NMDA agonists [18]. Several recent studies have shown that functional NMDA receptors are expressed in tumours [19], inhibiting intracellular calcium concentration and inactivating calcium-dependent cytosolic guanyl cyclase [20]. Thus, NMDA receptors play a critical role in tumour development, tumour growth and metastasis [21]. As a first messenger, calcium plays a central role in tumour cell progression by regulating various signalling cascades [22]. Calcium influx into the cytosol results in the calcium-dependent activation of second messengers, thereby activating proteins of various other groups including transcription factors; in turn, this activation influences further tumour cell behaviour [23]. The NFAT-dependent regulation of integrins was already described by Jauliac and colleagues in 2002 [24]. A more recent preliminary expression profile analysis carried out by our group showed the NFATc2- and Sp1-dependent regulation of integrin beta-3 in pancreatic cancer cells. Here, Sp1 acts as a transcriptional repressor of NFATc2 at the respective GC box of the promoter [25]. The present study shows that integrin beta-3 is expressed in the pancreatic cancer cell lines PaTu 8988t und PANC-1. Stimulation of the tumour cells with ketamine, s-ketamine or MK 801 increases the expression of integrin beta-3. Stimulation with ketamine and s-ketamine decreases the expression of NFAT transcription factors in the nucleus and increases the expression of these transcription factors in the cytoplasm. Thus, the application of ketamine and s-ketamine places NFATc2 in an inactive, dormant state [17]. Stimulation of pancreatic cancer cells with ketamine, s-ketamine or MK 801 significantly increases cell migration. Furthermore, our group has already shown that ketamine and s-ketamine inhibit both cell proliferation and apoptosis in pancreatic cancer cells [26]. Therefore, the following molecular biological mechanism may be conceivable (Figure 3): As NMDA receptor antagonists, ketamine, s-ketamine and MK 801 bind at the phencyclidine binding site inside the NMDA channel, thereby inhibiting the action of NMDA agonists. This inhibition decreases the intracellular calcium concentration, which, in turn, inactivates various signalling cascades, such as the calcium-calcineurin-NFAT pathway. As a result, NFATc2 remains in an inactive, dormant state in the cytoplasm and undergoes reduced dephosphorylation and nuclear displacement by calcineurin [27]. In the cell nucleus itself, control of the promoter is complex. Many proteins are involved, which act as transcription factors or co-factors, or both, and form the ‘transcription machinery’ together with the transcription factors NFATc2 and Sp1. Here, Sp1 acts as a transcriptional repressor of NFATc2 at the respective GC box of the promoter. Finally, the interaction of various and partly still unknown proteins increases the expression of integrin beta-3, which also increases cell migration.

## 5. Conclusions

Pancreatic ductal adenocarcinoma is one of the most aggressive malignant tumours in humans. Its oncogenic potential is primarily characterised by extremely rapid tumour growth and early-onset metastasis [28]. Novel therapeutic approaches target the effective modulation of specific signalling and transcription pathways [29]. The integrin inhibitor cilengitide (Merck), a cyclic pentapeptide, was also developed for this purpose. Cilengitide is considered a highly potent inhibitor of tumour-induced angiogenesis because it inhibits interaction with the extracellular matrix by binding to integrin αvβ3 and/or αvβ5 [30]. In a clinical study (Phase I/II) investigating recurrent and metastasised squamous cell carcinoma of the head and neck, cilengitide was tested in combination with cisplatin, 5-fluorouracil and cetuximab, but the additional administration of cilengitide did not yield any therapeutic advantage [31]. The basis and prerequisite for new therapeutic approaches in the context of a ‘target therapy’ is comprehensive knowledge about the carcinogenesis of the respective tumour entity. Therefore, further studies are required to identify the underlying disease mechanisms of pancreatic carcinoma. The identification and characterisation of cellular receptors as well as their extracellular activators will eventually help to establish new therapeutic options for the treatment of aggressive pancreatic cancer.

## List of Abbreviations:

DPBS: Dulbecco’s Phosphate-Buffered Saline
GC-Box: Regulatory Element Rich in Guanine and Cytosine
ITGB3: Integrine Beta-3
MK 801: Dizocilpine
NFATc2: Nuclear Factors of Activated T-cells
NMDA-Receptor: N-methyl-D-aspartate-Receptor
PCP: Phencyclidine
Sp1: Specificity Protein 1
TBS-Buffer: Tris-buffered Saline-Buffer
